# Addiction is Not a Brain Disease (and it Matters)

**DOI:** 10.3389/fpsyt.2013.00024

**Published:** 2013-04-11

**Authors:** Neil Levy

**Affiliations:** ^1^Florey Institute of Neuroscience and Mental Health, The University of MelbourneParkville, VIC, Australia

**Keywords:** addiction, disease, well-being, agency, dysfunction

## Abstract

The claim that addiction is a brain disease is almost universally accepted among scientists who work on addiction. The claim’s attraction rests on two grounds: the fact that addiction seems to be characterized by dysfunction in specific neural pathways and the fact that the claim seems to the compassionate response to people who are suffering. I argue that neural dysfunction is not sufficient for disease: something is a brain disease only when neural dysfunction is sufficient for impairment. I claim that the neural dysfunction that is characteristic of addiction is not sufficient for impairment, because people who suffer from that dysfunction are impaired, sufficiently to count as diseased, only given certain features of their context. Hence addiction is not a brain disease (though it is often a disease, and it may always involve brain dysfunction). I argue that accepting that addiction is not a brain disease does not entail a moralizing attitude toward people who suffer as a result of addiction; if anything, it allows for a more compassionate, and more effective, response to addiction.

Neuroscientists and other scientists involved in the study of addiction rightly see their work not merely as objective science but also as a compassionate project. It aims not only to elucidate the neuropsychological causes and correlates of addiction, but also to provide knowledge that can be applied in the treatment of people who are suffering. Even those whose work is far removed from the clinical coalface – those working on animal models of addiction, for instance – take their findings and those of their peers to have important implications for how we ought to respond to addicts. The elucidation of the neural underpinnings of addiction show that addiction is a disease that must be treated, not something for which addicts can be blamed. As Leshner (1997) has said, addiction is a brain disease, and it matters.

In this paper, I will argue that the slogan is, at best, misleading. Addiction is not best understood as a brain disease, though it certainly involves pathological neuropsychological dysfunction. Addiction is a disorder of a person, embedded in a social context. The neuroscientists and their allies have mistaken some necessary conditions of the disorder with the disorder itself^1^. Notwithstanding this claim, there is, nevertheless, a strong case for saying that addiction is often a disease. Restoring addicts to their social contexts does not require us to accept the view of addiction to which the neuroscientists oppose themselves, the moral model. Rather, we can situate the addict in a social context, and even recognize that judgments about disorder are partially normative, without abandoning an entirely naturalistic framework.

Neuroscientists embrace the brain disease model of addiction for an obvious reason: because they have made great progress in elucidating neural mechanisms and neuroadaptations that are correlated with, and undoubtedly causally involved in, addiction. Neuroscientists have identified a range of such changes, including (but not limited to) the longterm depression of reward circuitry and increased activity in antireward circuitry (Koob and Le Moal, 1997, 2008); alterations in the midbrain dopamine system (Volkow and Li, 2004); and in frontal regions involved in impulse inhibition (Goldstein and Volkow, 2002). However, there are neural changes associated with and causally involved in *all* behaviors. Establishing that this is true with regard to addiction therefore does not establish that it is a brain disease.

What would it take to show that addiction is a brain disease? The details of the neural correlates of addiction matter: addiction is a brain disease if these correlates are pathological and if that pathology is sufficient for the person to have a disease, in almost any accessible environment (I will say more about this condition later in the paper). I will argue that though there is a case for saying that the correlates of addiction are pathological, these correlates are not sufficient for the person to have a disease in some accessible environments. Regardless of whether the correlates are themselves pathological, the person has a disease only insofar as their functioning as an agent is impaired, and in many environments the correlates of addiction are not sufficient for impairment. Further, I will suggest, judgments about impairment are normative judgments, where the norms in question are not norms of brain functioning. However, since a normative judgment need not be a non-naturalistic judgment – the protestations of many to the contrary – accepting that the judgment that addiction is a disease is partially normative does not require accepting the moral model of addiction, as that model is standardly conceived.

## Are the Neural Correlates of Addiction Pathological?

If the judgment that addiction is a disease is unashamedly normative, and the norms in question are not norms of brain function, then addiction is not a brain disease. Addiction is a brain disease only if pathological deviations from norms of brain function are (in almost any accessible environment) sufficient for being impaired. Whether addiction is caused by pathological brain dysfunction is not as obvious, however, as it apparently appears to many scientists. There are scientific accounts of addiction according to which it does not involve any brain pathology at all. On the theories I have in mind, explaining addiction requires us to postulate *non-pathological* brain mechanisms.

Consider a *mismatch* account of addiction (Durrant et al., 2009). Mismatch accounts focus on a mismatch between our evolved capacities and dispositions, on the one hand, and the environment in which many people find themselves today, on the other. This kind of hypothesis seems a plausible (partial) explanation of the current obesity epidemic. Roughly, the idea is as follows: in the environment of evolutionary adaptiveness, calories were relatively scarce. It was therefore adaptive to develop dispositions to consume as much as possible of high-calorie (sweet or fatty) foodstuffs when they were available, given that these foods couldn’t be stored for long. Today, however, fatty and sugary foods are plentifully available, but we remain disposed to consume them beyond immediate and near-term need. Because we did not need to exercise self-control in the EEA with regard to these foods – just the opposite – we are reliant on limited, top-down, and domain-general mechanisms for self-control to resist overconsumption, and these mechanisms are relatively easily circumvented or exhausted. Hence the obesity epidemic. Just as in the absence of a domain specific reasoning mechanism for conditionals, we are required to use domain-general reasoning for their evaluation and we do predictably badly (Cosmides and Tooby, 1992), in the absence of substance-specific self-control mechanisms, we are thrown back onto our domain-general self-control mechanisms, and overconsumption is a likely result.

There have been attempts to develop mismatch accounts of mental illness. For instance, Murphy and Stich (2000) have hypothesized that depression might sometimes result from an overly (but not pathologically) sensitive relative status detector. Their proposal builds upon Nesse and Williams (1995) suggestion that depression may be an adaptive response to a fall in, or a failure to gain, status. Such failures trigger a disposition that focuses the individual inward (hence the rumination characteristic of depression) thereby encouraging them to identify unsuccessful social strategies and develop new ones, and causes them to withdraw from social contact which might trigger aggression on the part of dominant group members. Murphy and Stich propose that when the relevant social status detector is toward the more sensitive end of the normal distribution, it will be continually set off in contemporary societies in which we have constant opportunities to compare ourselves to high status individuals (celebrities, moguls, athletes, and so on) around the world. The result is the triggering of depression.

Consumption of mind-altering substances dates back thousands of years in human history, and very plausibly began in pre-history. Records of opium use date back nearly 6000 years (Booth, 1996); beer brewing dates back even further. However, it is unlikely that anyone had access to a sufficient quantity of such substances, over a sufficiently extended period of time, for these substances to generate serious problems until quite recently, when agriculture became widespread and humans became sedentary (Durrant et al., 2009); indeed, it is very likely that self-control with regard to them first came to be required only with the growth of cities. In these conditions, there was no selective pressure for human beings to develop a specific self-control mechanism with regard to these substances.

Today these substances are abundantly available, at least to some people and in some places (alcohol and tobacco for most people in developed nations; other substances for particular subgroups; addiction is correlated with lower socio-economic status, and addictive drugs tend to be more abundant in lower SES areas). Since we lack a substance-specific self-control mechanism, we are thrown back on domain-general self-control resources, and these resources are easily depletable (Baumeister and Vohs, 2007).

There is a salient difference between high-calorie food and drugs of addiction, though: it is plausible that evolution left us with dispositions to pursue the first but there is no evidence that we have innate dispositions to pursue the second (*pace* Sullivan and Hagen, 2002). However, the taste for drugs can be acquired; there is no reason why an acquired disposition need be weaker than an innate one. Moreover, even if the disposition to use drugs is less motivating, or motivates fewer individual actions, than the innate disposition to consume food, the harms associated with drugs accrue far more quickly, so a theory postulating a weaker disposition may explain the observed effects.

The mismatch theory does seem to go a long way to explaining the problems many people have with addictive substances. It also helps to explain why environmental factors that limit access to these substances are strongly protective. But the mismatch account clearly does not entail that addiction is a brain disease. The reason is simple: if the mismatch account explains (or, more plausibly, has a significant role in explaining) addiction, then it entails that addiction is explained by brain mechanisms *functioning as they are designed to* (Murphy and Stich, 2000). To the extent to which the mismatch account explains why addicts find it hard to resist addictive drugs, it entails that they are not suffering from a *brain* pathology at all.

If we are to show that addiction is a brain disease, we shall need to show that the underlying pathology is a pathology of the brain. We need to show that the brain is dysfunctional, in much the same way as medical scientists establish that an organ is diseased by showing that it is dysfunctional. The canonical example in medical science is heart disease: heart disease counts as a disease because it threatens to interfere with the function of the heart. The heart’s functional role is pumping blood; because heart disease interferes with that role, it is a disease.

Dysfunction accounts come in two varieties, corresponding to the two competing philosophical analyses of function. On a *selectionist* account, expounded most influentially by Millikan (1984), a dysfunction occurs when something fails to play the role for which it was selected in the evolutionary history of the organism. On the *systemic* account, developed by Cummins (1975), it is not the role that something played in evolutionary history that gives it its function; rather, it is the role it (or its homologs) actually plays in a system. I do not intend to try to settle the debate between these accounts. Rather, I shall focus on what the accounts have in common, arguing that neither entails that addiction is brain disease.

There are plausible theories which (partially) explain addiction and entail that the addict’s brain is dysfunctional. Suppose some kind of dopaminergic account of addiction is correct; suppose, that is, that addiction involves a pathology in the midbrain dopamine system. It is widely held that the midbrain dopaminergic system is a valuational system: it has the role of signaling the value of a resource to the organism and motivating the organism toward consumption of that resource. This view stems, in significant part, from important work of Schultz et al. In several experiments, Schultz et al. recorded the activity of midbrain dopamine neurons in monkeys performing various tasks that were rewarded with water or juice. In one experiment, monkeys learned that they would receive a reward if they pressed a particular lever, following a cue (Schultz et al., 1992). During the learning phase, the neurons responded strongly to the delivery of the reward, but once the task and the association between the cue and juice availability was learned, neurons responded when the cue was given, but not when the reward was delivered. Similarly, dopamine neurons in monkeys respond initially to the delivery of a reward predicted by a visual cue, but as the association between the cue and the reward comes to be learned, the response to the reward declines while the response to the cue predicting the reward increases (Sutton and Barto, 1998).

On the basis of this kind of evidence, many researchers have come to believe that the mesolimbic system is a reward prediction system (Montague et al., 1996; Schultz et al., 1997). It allows us to learn the value of a reward and the relationship between environmental cues and rewards. This function is obviously adaptive, since it plays a crucial role in guiding and motivating the organism in seeking out rewards, where “rewards” are goods needed for survival and reproduction.

However, addiction seems to involve dysregulation in this same midbrain dopamine system. Nearly all addictive drugs increase dopaminergic activity. Amphetamine, nicotine, cannabis, cocaine, and alcohol all either stimulate dopamine release or decrease dopamine reuptake. They thereby increase dopamine in the nucleus accumbens. Opioids increase dopamine indirectly, by influencing neurons that alter accumbal dopamine (Carter and Hall, 2012). Caffeine also increases extracellular dopamine in the nucleus accumbens (Solinas et al., 2002). The manner in which addictive drugs (and, in a very different way, gambling; see Ross et al., 2008) drive up the dopamine signal is widely thought to be central to explaining how addiction develops and why it is a chronic relapsing condition. For many addiction experts, addiction *is* a pathology of the dopaminergic system. In the common metaphor, addictive drugs “hijack” this system. That is, addiction crucially involves a *dysfunctional* mesolimbic system.

The reward prediction hypothesis seems to explain addiction by understanding it as a pathology of reinforcement learning. When the system is operating as it should, dopaminergic activation attenuates in response to *expected* reward. Dopamine response increases when the world is better than expected; when an expected reward is delivered, the world is exactly as expected and there ought to be no dopamine response. If drugs worked like natural rewards, we could expect them to trigger an initial dopamine response to consumption, but an attenuation of this response as consumption is repeated. At the same time, we ought to expect an increase in dopamine response to predictors of drug availability. Instead, what we find is dopamine response to predictors of drug availability *and* – because drugs of addiction drive up the dopamine response by their chemical action – continuing dopaminergic activity at consumption as well. In effect, the dopaminergic system responds to drugs with the signal that consumption is better than expected. It does so *every time* the drug is consumed. The addict cannot learn the reward value of the drug, because the system for reward value learning is dysfunctional. On every occasion the drug is consumed, the dopaminergic system reports that the drug is more rewarding than expected. The result is pathological learning; the system treats the drug as of ever increasing value.

It should be noted that there are rivals to the reward prediction interpretation of mesolimbic dopaminergic activity. Berridge, 2007 suggests that the role of dopamine is incentive salience, not learning. Berridge points out that learning about the relationship between a stimulus and a reward can occur without dopamine. In mice genetically engineered to be unable to synthesize dopamine, normal learning seems to occur. It also occurs in mice that have virtually no mesolimbic dopamine due to neurochemical lesioning. Further, activation in the ventral pallidum, downstream of the mesolimbic dopamine system, is stronger in response to a second, redundant, predictor of reward than in response to the first. Since the second predictor adds no new information, we ought to expect a smaller response to the second predictor than to the first if the dopamine system was itself a reward prediction system.

For Berridge (2007; Holton and Berridge, forthcoming) addiction is a pathology of incentive salience and not reward prediction. It does not involve pathological learning; rather it involves pathological “wanting.” We can leave this dispute to one side. For our purposes what matters is what the researchers agree upon: that the dysfunction in the dopaminergic system either is, or is reflective of, a dysfunction of a system that evolved to play (or normally plays) a specific role in behavior, whether that is learning or incentive salience. On either story, we could understand the system that is awry, whether it is the midbrain dopamine system or something upstream of that system, as representing something about goods in the external world, such as how valuable they are to the organism. On either story, addiction causes a misrepresentation and for that reason it is a pathology. It is a pathology because the system was selected to play, or actually plays, a particular role in the psychology of animals like us, but it is no longer playing that role in addicts, at least in response to drugs and cues predictive of drug availability.

Both these dysfunction accounts, together with plausible hypotheses concerning the neural correlates of addiction, entail that addiction involves a neuropsychological dysfunction. However, neither account entails that addiction is a brain disease. I claimed earlier that addiction is a brain disease only if two conditions are satisfied: its neural correlates are pathological, and that pathology is sufficient for the person to be suffering from a disease in almost any accessible environment. This second condition is necessary to rule out conditions in which the appropriate response to suffering is to alter the environment and not to “treat” the person. Consider homosexuality. It remains an open question whether the neural correlates of homosexuality are adaptive. Wilson’s (1975) conjecture, for instance, according to which homosexuality exists today due to frequency dependent selection maintaining the relevant genes at some low frequency in our ancestral population, might yet be proven correct. If this hypothesis, or another one that entails that homosexuality is adaptive (or at least not maladaptive) is false, however, it does not follow that homosexuality is a disease, not even if homosexuals suffer in homophobic societies. The conjunction of causation by dysfunction plus impairment is not sufficient for disorder, when the impairment is due to social conditions that can relatively easily be altered; that is, when the alterations necessary to remove the impairment are not alterations we have good reason to refuse to make (because they would impose significant costs on third parties, for instance)^2^. I express this claim by saying it is a necessary condition of a condition being a disease that it causes suffering in almost any accessible environment. If it is the case that there is an accessible environment – where accessibility is a function not merely of physical possibility, but also of the costs (economic, social, moral) of actually accessing that environment – in which a dysfunction does not cause an impairment, then the dysfunction is not sufficient for a disease.

Apparent counterexamples to this account are, I claim, only apparent. Consider peanut allergy^3^. It is certainly possible to alter the environment of sufferers such that they do not suffer any impairment. That fact entails that if such alterations are sufficiently cheap, peanut allergy is not a disease. This seems to be contrary to standard medical usage (ICD-10, for instance, has an appropriate category for peanut allergy). Standard medical use notwithstanding, however, I maintain that if it is true that there are accessible environments in which a peanut allergy does not cause any impairment, then it is not a disease (perhaps the intuition that it is a disease is partially due to the fact that avoiding peanuts is, right now, far from costless, since the burden is placed on individuals to carefully monitor their diet in an environment in which many products and dishes contain traces of nuts sufficient to trigger the allergic reaction). Compare a peanut allergy to dyslexia. Dyslexia may have a genetic basis, but it seems wrong to say that our hunter-gatherer ancestors suffered from dyslexia prior to the invention of writing. Rather, dyslexia seems to be a disease only in a society in which reading is sufficiently important for reading problems to count as a disability (Buchanan et al., 2000, p. 123). Now, if it is true that dyslexia was not a disease in the pre-literate past, because it did not cause an impairment in those who (in some attenuated sense) suffered from it, then it seems that if it were possible costlessly to alter the environment so that it did not cause an impairment in sufferers today, it would not count as a disease today. It would be analogous to homosexuality, inasmuch as it would be incumbent on us to eliminate the suffering it causes by altering the environment. The example of peanut allergy also seems to be closely analogous, and therefore I maintain that it does not constitute a counterexample to the account offered^4^.

According to this account, addiction is not a brain disease, because it is sometimes not a disease at all. While the claim that the dopaminergic system is dysfunctional in addicts is plausible, a dysfunction of this kind is not sufficient for impairment in many accessible environments. The misrepresentation identified is at a subpersonal level, but an agent suffers from a pathology of the mind only when there is personal-level problem. Mental illness is quite plausibly identified with a defect of rationality of some kind (Graham, 2010), and a subpersonal misrepresentation is not a defect of rationality. It is quite possible for *mechanisms* to misrepresent while *agents* properly represent; once someone is acquainted with a particular visual illusion this might be true of her on future encounters with it. Of course, a subpersonal misrepresentation might in some cases straightforwardly cause a personal-level misrepresentation, but that doesn’t seem to be – straightforwardly – the case in addiction. If agents accepted the valuation placed on the drugs to which they are addicted by subpersonal mechanisms, they would not want to give up, and a range of facts about them would be inexplicable (why they often, though not always, say they want to give up; why they expend significant resources in an apparent attempt to give up (Ross et al., 2008); why spontaneous recovery is so common).

On the Holton and Berridge (forthcoming) view, addiction involves intense cravings as well as misrepresentations; the midbrain dopamine system for them is the system that generates the cravings rather than itself a representational system. This addition to the account might go some way toward explaining how addiction is a personal-level defect: the agent experiences these cravings no matter how she judges, and is therefore motivated to act. However, even with this addition it seems that the hypothesized dysfunctions fall short of a pathology, because there are accessible environments in which the agent will not suffer from any defect of rationality or impairment of agency. There are two reasons for this. The first is that the dysfunctions identified are not sufficient for the experience of cravings. Cravings for drugs are heavily cue-dependent, and the cues that trigger these cravings are avoidable (how easily the agent may avoid them will differ from person to person, depending on their learning history; in any case, for some agents with the relevant dysfunction, there will be accessible environments in which cues are avoidable).

Second, though cravings are unpleasant, experiencing them seems to fall very far short of any kind of mental illness or pathology. Subpersonal over-valuation of drugs plus intense cravings are not sufficient for the person to suffer from a defect of rationality. Nor are they sufficient for the person to suffer from a sufficiently serious impairment of agency or of their ability to pursue a worthwhile life. The neuroadaptations characteristic of addiction are longlasting; it is for this reason that the Alcoholics Anonymous slogan “once an alcoholic, always an alcoholic” has more than a grain of truth to it. Yet plainly the former heavy drinker or drug taker who has been abstinent for many years need not be suffering from any impairment (though she may have a vulnerability to suffering an impairment). All by itself, this fact shows that the neuropsychological dysfunction underlying addiction is not sufficient for disease. Indeed, with regard to some addictions some individuals who satisfy the dysfunction condition suffer no impairment despite continuing to take the drug. Whether this is true will vary depending on the drug, the consumption method, and (importantly) the ability of the agent to access the drug safely and reliably. Most people addicted to caffeine suffer no impairment. More controversially, some individuals addicted to benzodiazepines or to nicotine delivered by “e-cigarette” may suffer no impairment of rationality, of agency or of the capacity to pursue a worthwhile life. Even some heroin addicts, with the resources to obtain heroin from safe sources, may not suffer harms significant enough to plausibly constitute an impairment of their agency or their ability to pursue a good life.

It will not help the defender of the brain disease account to add the other neural correlates associated with addiction into the mix. Consider the chronic deviation from reward set point identified by Koob and Le Moal (2008). The allostatic state they postulate is the result of the brain adapting to drug ingestion. So long as the drug is reliably available, the person will suffer no ill-effects from this neuroadaptation^5^. Rather, the anhedonic state from which individuals suffer is associated with chronic abstinence. Identifying the pathology with this unpleasant state entails, counter-intuitively, that the abstinent addict suffers from a pathology but the addict who is using does not.

Other neuroadaptations characteristic of addiction are more plausible candidates for an agency-impairing pathology. Dysfunctions in mechanisms involved in self-control can be expected to impair agency under a range of conditions. However, in many environments and for many individuals, the defect is not so significant as to entail an impairment of agency or rationality. Rather, in supportive environments, where the agent is buffered from many demands by social support, this impairment is fully compatible with pursuing a good life.

If my claim that the neural correlates of addiction do not cause impairment in all accessible environments is true, addiction is *not* a brain disease in the way in which the other conditions Leshner (1997) cites are. Stroke, schizophrenia, and Alzheimer’s disease cause significant defects of rationality and agency in almost any environment; though it might be possible to imagine environments in which some of these conditions did not cause impairment, such environments are not genuinely accessible (the costs of maintaining them would be prohibitive, to begin with). Addiction differs from paradigm brain diseases in that its correlates do not cause impairment across all, or nearly, accessible environments. For some conditions that cause suffering, neural correlates are sufficient to cause an impairment and for some they are not; only those which fit into the former class count as brain diseases. Addiction fits into the latter class because, with the possible exception of some deficits that are likely relatively minor (such as somewhat impaired self-control mechanisms), addiction only causes impairments in certain social environments, and social environments in which addiction would not cause any significant impairment are accessible.

These remarks suggest that if addiction is properly understood as a disease or pathology, it is not *just* because it involves neuropsychological dysfunction. Rather, capturing the manner in which it is pathological requires that we adopt an explicitly normative account of pathology, according to which someone suffers from a pathology when and only when they are subject to significant impairments of agency and consequently of the ability to pursue a good life. In the absence of such impairment, the person who ingests drugs and undergoes the neural changes associated with longterm drug use does not suffer from a disease.

The forgoing remarks ought not to come as a surprise: they amount to nothing more than the claim that addiction must fit the influential two-stage model of disease or disorder. On the two-stage model, an individual suffers from a disorder only if they experience a biological dysfunction and that dysfunction is harmful, where the judgment of harm is made by reference to social norms of flourishing (Wakefield, 1992; Murphy, 2006). Biological dysfunction may be a necessary condition of being a disorder. But this necessary condition is not a sufficient condition, and addiction is not a brain disease. Rather, *when* it is a disease, it is a disease that essentially involves brain dysfunction.

## Why it Matters that Addiction is Not a Brain Disease

The claim that mental illness partially, but essentially, involves some deviation from norms does not entail accepting the moral model. It does not entail that addicts are to blame for their addiction. This ought to be obvious, since the account emphasizes that addiction may not count as a disease because the suffering it causes is very largely due to social conditions that are, in some sense, optional; clearly the addict is not herself responsible for these conditions. Nor does the account entail that addiction is not real, or that the suffering involved is not genuine. There may be a fact of the matter whether and when addicts suffer from a genuine impairment of agency. There are a variety of realist accounts of what constitutes a good life. Addiction may be a normative failing but if these accounts are correct it is not a normative failing *rather* than a naturalistically explicable disorder. Rather, it is a normative failing *because* of the kind of naturalistically explicable disorder it is.

The forgoing remarks are important, because they help us to recognize that the insistence that addiction is a brain disease is merely one way we can avoid both the crass moralism of those who blame addicts and a facile relativism about disorders. Addiction is not a brain disease, but there is a good case for saying that it is, nevertheless, a disorder which may require treatment (which may be medical or psychiatric, though other kinds of treatment may be appropriate in addition or instead), for which the sufferer is not to blame and the sufferer from which is an appropriate recipient of compassion.

To that extent, my claim that addiction is not a brain disease may seem to change nothing, compared to the situation that would prevail were the scientists’ claim that it is a brain disease to be accepted. Though the overlap between the two accounts is important, there are some important differences.

The claim that addiction is not a brain disease allows us to resituate the addict in her social environment (Levy, 2007). She suffers from a disorder only insofar as her brain is dysfunctional in certain ways *and* prevailing social conditions make it likely that she will suffer from a defect of rationality or an impairment of agency as a result. This may be due to the fact that she lacks the resources to remove herself from environments in which she frequently encounters the cues that trigger cravings in her, and in which her self-control resources are depleted by constant demands, stress, and poor nutrition. It may be due to the fact that she lacks access to goods that compete with the attractions of the drug. The facts that explain her addiction, and the facts that explain her suffering (and the suffering she causes to others) are partially facts about her, and partially facts about the environment in which she is embedded. Moreover, the facts about her that explain her addiction and the associated suffering are themselves mediated by her environment (and some – and only some – of the facts about her environment are mediated by her).

Responding appropriately to addiction, as well as allocating blame between addict and other actors, requires us to be sensitive to these facts^6^. Addiction is a pathology that involves neuropsychological dysfunction, and it may be appropriate to respond to it by treating this dysfunction (pharmacologically, for instance). But addiction is a pathology only because of the addicts’ social embeddedness, and it may equally be appropriate to respond to it by altering the social conditions that cause and sustain it, or which cause and sustain the impairments it gives rise to. If we are to understand addiction and respond appropriately to it, we must not focus on just the addicted individual herself, much less her brain. Our focus must be on her, in her social setting. Inevitably, that entails that *we* must ourselves come under scrutiny; perhaps we need to change as much as she does.

## Conflict of Interest Statement

The authors declare that the research was conducted in the absence of any commercial or financial relationships that could be construed as a potential conflict of interest.

## Footnotes

^1^It should be noted that Leshner (1997, p. 46) himself recognizes that addiction is not *just* a brain disease; rather he claims that it is “a brain disease for which the social contexts in which it has both developed and is expressed are critically important.” For Leshner, addiction is a brain disease in a social context, just like (to cite his own examples) stroke, schizophrenia, and Alzheimer’s disease. My claim is that addiction is *not* a brain disease like the other conditions Leshner cites; it has crucial features that make it different from stroke, schizophrenia, and Alzheimer’s.

^2^Suppose, however, that impairment is caused by a dysfunction plus social conditions we cannot alter, or altering which would be prohibitively costly, but those social conditions are unjust. Suppose, for instance, that some kind of biological dysfunction caused an impairment only because people were repulsed by it; would such a condition count as a disease (I thank Jerome Wakefield for raising this question)? I think we should count any dysfunction that causes impairment as a disease when the impairment cannot be (for practical purposes) avoided. This entailment of my account might strike some as counterintuitive; perhaps the impression might be softened by pointing out that in cases like this, we might owe sufferers from the impairment some special duty of recompense or compensation. Gillet and Huang (forthcoming) argue the case for such a view with regard to psychopathy.

^3^Thanks to Richard Holton for pressing me on this issue; the suggestion that peanut allergy is a counterexample to the account of disease offered here is due to him.

^4^The example of dyslexia well illustrates how “accessibility” is a partly normative notion: an environment counts as accessible if it is not merely physically possible for an individual suffers access it, or for a whole society to adopt its norms, but it is reasonable to expect the person or society to take these steps. There may continue to be extant cultures in which dyslexia does not cause an impairment, because literacy is not a benefit to members. However, for the vast majority of sufferers these cultures are extremely hard to access: barriers to entry to these cultures (learning a new language and a new way of life) are high, newcomers may not be easily accepted and people originally enculturated into a different way of life might find the new culture very unsatisfying. In addition, members of these cultures may be inadequately fed, may lack access to clean drinking water and to health care. These facts entail that it would be unreasonable to expect most dyslexics to avoid impairment by accessing these cultures, even if there is some sense in which some of them could do so; for closely analogous reasons, it would be unreasonable to expect developed societies to adopt the ways of life of such cultures. I thank Jerome Wakefield for pressing me on this issue.

^5^Because reliable availability of the drug enables the addict to maintain homeostatic equilibrium, addiction seems to be a counterexample to Roe and Murphy’s (2011) claim that it is a necessary condition of having a disorder that the mechanisms designed to maintain homeostasis be disordered. An addict may suffer an impairment, caused by a dysfunction of the dopaminergic system, and yet be able to maintain homeostasis.

^6^The claim that an adequate response to addiction requires the treatment of the addict in her context, and therefore addressing the social factors that not only cause suffering but which also play a causal role in sustaining the behavior, has been sensitively developed by Hanna Pickard (see Pickard, 2012; Pickard and Pearce, forthcoming).
